# The effectiveness of acupuncture at Yaotongdian (EX-UE 7) for acute lumbar sprain: A protocol for a systematic review and meta-analysis

**DOI:** 10.1097/MD.0000000000024440

**Published:** 2021-01-29

**Authors:** Pan Yue, Juan Zhong, Jiajun Huang, Zhaoxi Lan, Sen Zhong

**Affiliations:** a Hospital of Chengdu University of Traditional Chinese Medicine; b School of Clinical Medicine, Chengdu University of Traditional Chinese Medicine; c Affiliated Sport Hospital of Chengdu Sport University, Chengdu, Sichuan Province, P.R. China.

**Keywords:** acupuncture, acute lumbar sprain, extraordinary acupoint, protocol, systematic review, Yaotongdian (EX-UE 7)

## Abstract

**Background:**

Acupuncture at Yaotongdian (EX-UE 7) is increasingly used in acute lumbar sprain (ALS), and many studies have published that it is effective in the treatment of ALS. However, it is controversy if acupuncture at (EX-UE 7) can provide an evidence-based clinical benefit in the ALS population.

**Methods:**

We will go through 8 databases, and conduct a systematic review of acupuncture on (EX-UE 7) and health-related outcomes in ALS patients according to the Preferred Reporting Items for Systematic Reviews. The primary objective is to assess the impact of Preferred Reporting Items for Systematic Reviews and Meta-Analyses guidelines on clinical outcomes relevant to ALS patients, such as effective rate, life-quality evaluation, and adverse events. Cochrane Risk Assessment Tool will be used to assess the quality of eligible studies according to the Cochrane handbook.

**Results:**

The results of this systematic review will provide a synthesis of current evidence of Acupuncture at Yaotongdian (EX-UE 7) and we have a specific opportunity to determine the efficacy and safety of it.

**Conclusion:**

This study will explore whether or not acupuncture on (EX-UE 7) can be used as one of the non-drug therapies to prevent or treat ALS.

**Registration number:**

10.17605/OSF.IO/29QV7 (https://osf.io/29qv7/)

## Abstract 2

Supplemental Digital Content is available in the text

## 1 Introduction

### 1.1 Description of the condition

Acute lumbar sprain (ALS) refers to acute injury of the lumbar soft tissue of muscles, ligaments, or facet joints due to an inappropriate lumbar activity, such as moving or lifting objects of excessive weight or in an improper body posture, when the lumbar muscles are contracted powerfully. The patients may present with low back pain and impaired movement, influencing their normal life, and work.^[1]^ The incidence of this musculoskeletal injury is significantly higher among some special groups of people such as athletes and ship crew attributed to their routine training or work.^[2,3]^

### 1.2 Description of the intervention

Conventional treatment for ALS generally consists of drug and non-drug therapies. Medications for ALS mainly include anti-inflammatory analgesics such as ibuprofen and celecoxib, and centrally-acting muscle relaxants such as chlorzoxazone and eperisone, but both have the possibility to cause adverse reactions like gastrointestinal tract injury or hepatic and renal damage.^[4]^ As for non-drug therapies, bed rest and waist support brace are applied most, which may be time-consuming or easy to result in low back stiffness.^[5]^ Physical therapy has been advocated as an effective treatment for acute low back pain, but disagreement exists regarding its benefits, and international guidelines contain conflicting recommendations for manipulation and exercise therapy.^[6]^ Consequently, alternative therapies play an important role in the treatment of ALS, and non-drug approaches of traditional Chinese medicine (TCM) such as acupuncture and Chinese massage were applied most.^[7]^ Many studies have already shown that acupuncture stimulation at a variety of relevant acupoints have therapeutic effects on ALS.^[8]^ In the latest TCM guidelines of orthopedics and traumatology, 8 specific acupoints are recommended for acupuncture stimulation in the treatment of ALS, including 5 local acupoints (acupoints distributed on the low back area): Shenshu (BL 23), Dachangshu (BL 25), Zhishi (BL 52), Mingmen (GV 4), and Yaoyangguan (GV 3), and 3 distal acupints (acupoints distributed far away from low back area): Weizhong (BL 40), Chenshan (BL 57), and Kunlun (BL 60).^[9]^ Six of them belong to the Bladder Meridian of Foot-Taiyang and the other 2 belong to the Governor Vessel. However, the search for more effective acupoints in the treatment of ALS never stops. Apart from other meridian acupoints, many recent studies turned to extraordinary acupoints such as Yintang (EX-HN 3),^[10]^ Yaotongdian (EX-UE 7),^[11]^ and Dong's Extraordinary Point.^[12]^

### 1.3 How the intervention might work

**Figure 1:**
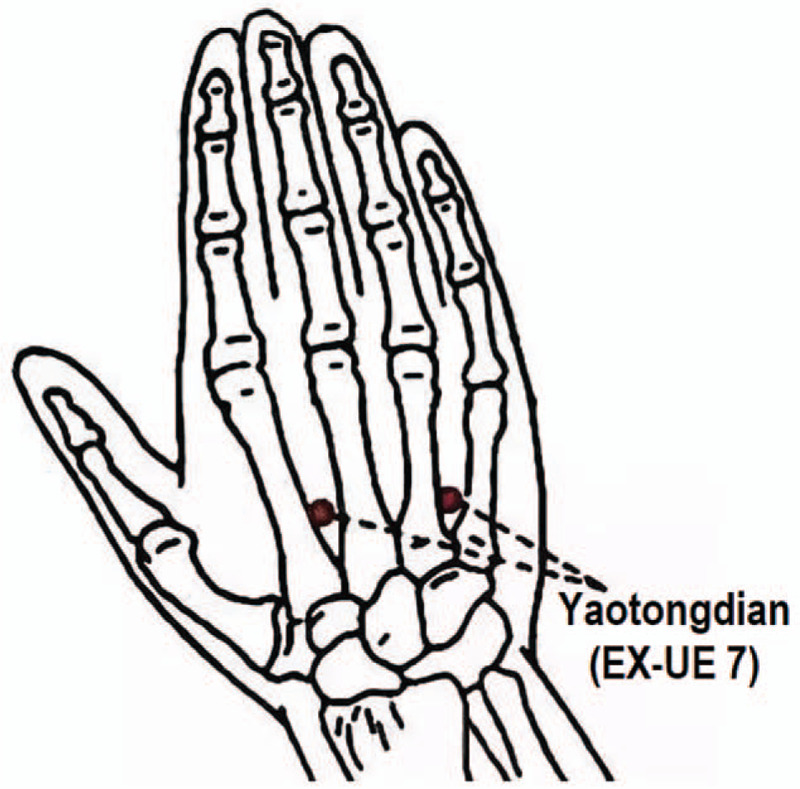
Location of Yaotongdian (EX-UE 7).

Yaotongdian (EX-UE 7) belongs to the extraordinary acupoints on the upper extremities. It is a pair of points located on the dorsum of each hand, one between the 2^nd^ and 3^rd^ metacarpal bones, and the other one between the 4^th^ and 5^th^ metacarpal bones, and both are at the midpoint between the transverse wrist crease and metacarpophalangeal joint (Fig. 1). They have become a pair of empirical stimulation points for low back pain in TCM since 1600 s, first named Weiling and Jingling by Yunlin Gong, an imperial physician in the Ming dynasty.^[13]^ Named after its function, “Yaotongdian” is exactly “lumbar pain points” in Mandarin, and its chief indication is just ALS.^[14]^ Hence it is often applied in the treatment of lumbar injuries and diseases, including ALS.^[15]^ There are randomized controlled trials (RCTs) indicating that acupuncture at Yaotongdian combined with other treatment had better analgesic effect and could improve the cure rate or shorten the course of ALS.^[16,17]^

### 1.4 Why it is important to do this systematic review and meta-analysis

Among the extraordinary acupoints, Yaotongdian were utilized and studied most in the treatmeant of ALS because of its own distinctive history and feature. However, there is no critically appraised evidence as systematic review or meta-analysis of the effectiveness and safety of acupuncture at Yaotongdian for ALS, and Yaotongdian has not become a recommended acupoint for ALS in the present TCM guidelines of orthopedics and traumatology. If acupuncture at Yaotongdian proved to be truly effective and safe, it could become a new recommended distal acupoint in the TCM guidelines for more physicians and acupuncture practitioners to utilize in the treatment of ALS, and may trigger further researches on the mechanisms of its analgesic and curative effects.

### 1.5 Objectives

In this study we intend to systematically review published RCTs about acupuncture at Yaotongdian (EX-UE 7) in the treatment of ALS and conduct a meta-analysis about its efficacy and safety. We look forward to providing sound evidence as reference basis about this unique extraordinary acupoint for physicians and acupuncture practitioners as well as TCM guidelines.

## 2 Methods

This study had been registered in https://osf.io/29qv7/ The registration number is: Identifier: DOI: 10.17605/OSF.IO/29QV7 This meta-analysis will be based on the preferred reporting items for the systematic review and meta-analysis of the project.^[18]^

### 2.1 Inclusion criteria for study selection

#### 2.1.1 Type of studies

All the RCTs to explore the efficacy and safety of acupuncture at Yaotongdian in the treatment of ALS will be included. Cross-trials, quasi-RCTs, case reports, observation studies, animal studies, repeatedly published studies, and studies did not have access to complete data will be excluded. Language and time of publication will not be restricted. If we are unable to find at least 5 eligible RCTs for the systematic review, we will broaden our inclusion criteria to include semi-randomized control studies, non-randomized studies of acupuncture at Yaotongdian (EX-UE 7) in ALS patients using the Cochrane Effective Practice and Organization of Care approach to categorize the types of studies.^[19]^

#### 2.1.2 Types of participants

Participants who meet the diagnostic criteria of ALS will be included, regardless of their gender, age, and race. However, ALS combined with lumbar disc herniation, lumbar spondylolisthesis or other orthopedic diseases were excluded. Diagnostic criteria will be based on the Clinical Guidelines for Diagnosis and Treatment of Orthopedics^[20]^ and Guidelines for Diagnosis and Treatment of Common Diseases of Orthopedics and Traumatology in TCM.^[9]^

#### 2.1.3 Types of interventions and controls

Interventions include acupuncture at Yaotongdian (EX-UE 7) with other complementary therapies or conventional treatment in the observational group. In the control group interventions only include other complementary therapies or conventional treatment. All the conventional treatment and complementary therapies are based on the clinical and TCM guidelines.^[9,20]^ However, studies that compare the efficacy of different forms of acupuncture at Yaotongdian (EX-UE 7) will be excluded.

#### 2.1.4 Types of outcome measures

The primary outcome is the clinical cure rate (Clinical cure is defined as complete relief of lumbar pain and normal range of motion).^[21]^ The secondary outcomes include visual analog score, the lumbar range of motion, and Oswestry disability index.

### 2.2 Search methods for identification of studies

#### 2.2.1 Data sources

PubMed, EMBASE (Excerpta Medical Database), the Cochrane Library, the Chinese Cochrane Centre's Controlled Trials Register platform, the Wanfang Chinese digital periodical and conference database, China National Knowledge Infrastructure database, and the VIP Chinese Science and Technique Journals Database will be searched by our authors for relevant literature. The data will be searched in English and Chinese databases from their inception to December, 2020.

#### 2.2.2 Other search resources

Chinese Clinical Trial Registry Center will also be screened for ongoing trials. We will also review the references of included manuscripts to identify any information about missed trials. We will contact the author if we cannot clearly identify information from the data.

#### 2.2.3 Search strategy

We will employ a broad electronic search strategy in Supplemental Digital Content (Appendix A, http://links.lww.com/MD/F599).

### 2.3 Data extraction, quality and validation

#### 2.3.1 Study selection and inclusion

Researchers will import the literature retrieved to the Endnote X7 and eliminate the duplicate data. All titles and abstracts returned using the search strategy above will be screened by 2 independent investigators (JZ, ZXL) in line with our advanced inclusion criteria. And then, the full text of the entire study will be reviewed by 3 authors for analysis. Any differences will be resolved by consensus. Finally, another study member will resolve the inconsistencies and check the final literature that will be included.

#### 2.3.2 Data extraction and management

**Figure 2:**
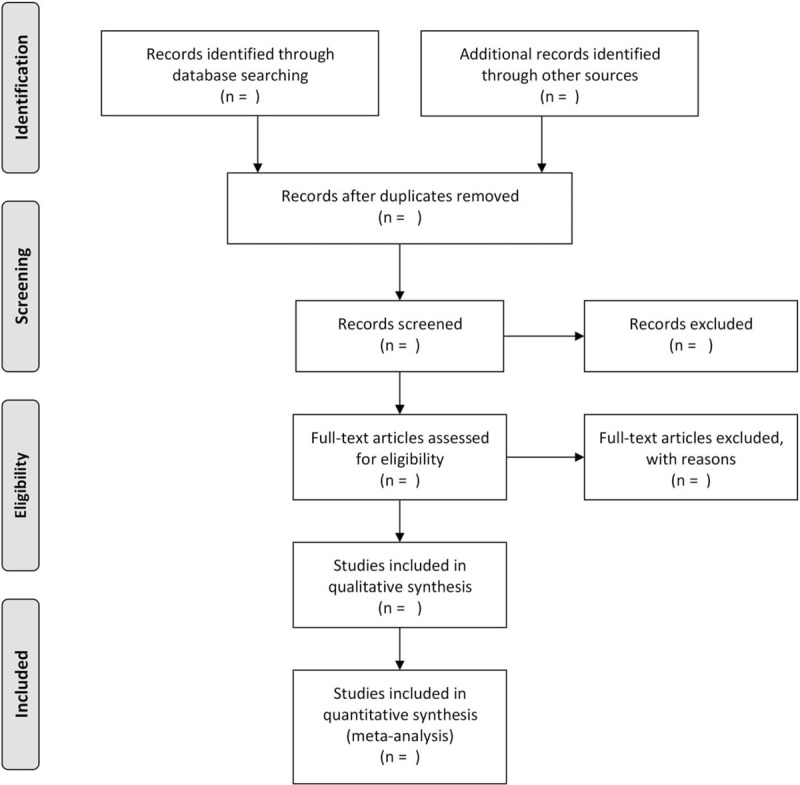
Flow diagram of study selection process.

The raw data from the papers will be extracted by 3 authors (PY, SZ, JJH) and will include: author details, publication information, sample size, and original study design information, such as intervention and comparison (dose, route, and time), outcome measures, and follow-up information. Catgut brand information will be also extracted from us if possible. All extracted data will be verified by a second investigator to ensure accuracy and completeness. All outcome variables will be collected, regardless of the number of studies that the outcome assessed. If conflict, arbitration will be conducted through discussion or through the third reviewer (JJH). Preferred Reporting Items for Systematic Reviews and Meta-Analyses diagram (Fig. 2) based on the search strategy and eligibility assessment to show the flow of included and excluded studies will be developed by us.

### 2.4 Assessment of risk of bias

The methodological quality of the included RCTs will be assessed based on the instrument developed in the Cochrane Handbook for Systematic of Interventions by 3 investigators. The tool evaluates studies based on 7 criteria:

1) randomization generation,
2) allocation concealment,
3) blinding of outcome assessors,
4) blinding patients/study personnel,
5) incomplete outcome data (that is, lost to follow-up),
6) selective outcome reporting, and
7) other risks of bias.

We will define other bias as trials which may be sponsored by acupuncture at Yaotongdian (EX-UE 7) manufacturers, and in which baseline characteristics are not similar between the different intervention groups. We will also assess publication bias by examining funnel plots if there are 10 or more trials reporting the primary outcomes.

### 2.5 Quantitative data and statistical methods

#### 2.5.1 Quantitative data synthesis

Review Manager (RevMan, Cochrane) software version 5.3 will be applied to pool our data to perform the meta-analysis. Measurements of dichotomous data will to be expressed as relative risks along with 95% confidence intervals; for continuous data, mean difference, 95% confidence intervals will be adopted, and *P* < .05 will be defined as statistically significant.

#### 2.5.2 Assessment of heterogeneity

In our review, I^2^ values will be used to assess inter-study heterogeneity. When I^2^>75%, considerable heterogeneity will be conformed, whereupon a random effects model will be applied. We will pool trials when the intervention form of those studies is adequately similar. Specific subgroups will be analyzed according to similar intervention forms or similar design.

#### 2.5.3 Assessment of reporting bias

If a sufficient number of studies are available (at least 10 studies), we will attempt to assess publication bias using a funnel plot.

#### 2.5.4 Subgroup analysis and investigation of heterogeneity

If there is a significant heterogeneity in the included trials, we will conduct subgroup analysis based on the type of disease, differences in treatment frequencies and follow-up duration will also be included.

#### 2.5.5 Sensitivity analysis

If the test for heterogeneity *P* value is less than .1 after performing the subgroup analysis, the sensitivity analysis will be conducted to evaluate the robustness of our results. The meta-analysis will be repeated after omitting the low-quality studies. Moreover, we will also assess whether the statistics model (random-effects model and fixed-effects model) will affect the current results.

#### 2.5.6 Grading the quality of evidence

We will apply the Grading of Recommendation Assessment, Development, and Evaluation method to evaluate the level of confidence in regards to outcomes. Two independent reviewers will conduct the assessment. In most cases, disagreements were resolved by discussion. If disagreement remained after discussion, a third reviewer will be consulted before taking the final decision on the disagreement.

## 3 Discussion

ALS is a common orthopedic disease frequently occurs in the young and mid-aged people, influencing their normal life and work. Medications for ALS do not have much to offer except NSAIDs and skeletal muscle relaxants which may lead to unpleasant or even serious side effects, while TCM has some superiority in its treatment. Many clinical studies have proved the curative effect of acupuncture in the treatment of ALS,^[22]^ and as a non-drug alternative therapy acupuncture is welcomed and widely utilized especially among special groups of people such as athletes and soldiers who are sometimes restricted in drug-use.^[23,24]^ Some of the effective meridian acupoints have already been recommended for ALS in the TCM guidelines, but there are not any recommended extraordinary acupoints maybe because of the lack of solid evidence as reference. Acupoints are generally divided into 3 categories: meridian acupoints, extraordinary acupoints, and Ashi acupoints. Extraordinary acupoints are empirical points with specific names and definite locations, but not as yet assigned into the 14 meridians. Verified repeatedly through clinical practice in the TCM history, extraordinary acupoints have fewer indications but excellent therapeutic effects in treating certain diseases.^[14]^ Therefore, they have long been widely used in the clinical practice though their mechanisms remain unknown.^[25]^ Yaotongdian eventually became the only extraordinary acupoint specifically nominated for low back pain after being verified over and over again in the past centuries. Many recent studies have also shown that acupuncture at Yaotongdian alone or combined with other therapies have curative effect in the treatment of ALS.^[26,27]^ Besides, unlike those recommended local acupoints on the low back area which have to be punctured in the prone position with needles of 2 to 3 cun (5 to 7.5 cm) in length or some distal acupoints with important nerves and blood vessels underneath such as Weizhong (BL 40), Yaotongdian is on the dorsum of each hand and its needling operation is relatively simple and safe: just puncturing obliquely 0.5 to 0.8 cun (1.5 to 2 cm) toward the center of the palm^[14]^ and patients need not take off their clothes or lie on their front. This systematic review and meta-analysis will help determine the potential benefits and harms of acupuncture at Yaotongdian in the treatment of ALS. The findings of this study may provide sound evidence as reference basis for the TCM guidelines and for more physicians and acupuncture practitioners to utilize Yaotongdian as an acupuncture point in the treatment of ALS which would benefit more patients in the future. The findings may also trigger further researches on the mechanisms of its analgesic and curative effects.

## Author contributions

**Conceptualization:** Pan Yue, Zhaoxi Lan.

**Data curation:** Juan Zhong, Sen Zhong.

**Formal analysis:** Jiajun Huang.

**Funding acquisition:** Sen Zhong.

**Investigation:** Sen Zhong.

**Methodology:** Zhaoxi Lan.

**Resources:** Jiajun Huang.

**Supervision:** Jiajun Huang, Zhaoxi Lan, Sen Zhong.

**Writing – original draft:** Pan Yue, Juan Zhong.

**Writing – review & editing:** Pan Yue.

## Abbreviations

Abbreviations: ALS = acute lumbar sprain, EX-UE 7 = extraordinary acupoint 7 on the upper extremities, RCTs = randomized controlled trials, TCM = traditional Chinese medicine.
